# Music Therapy for Children With Autistic Spectrum Disorder and/or Other Neurodevelopmental Disorders: A Systematic Review

**DOI:** 10.3389/fpsyt.2021.643234

**Published:** 2021-04-09

**Authors:** Hanna Mayer-Benarous, Xavier Benarous, François Vonthron, David Cohen

**Affiliations:** ^1^Department of Child and Adolescent Psychiatry, APHP.SU, Pitié-Salpêtrière Hospital, Paris, France; ^2^Department of Child and Adolescent Psychopathology, Amiens University Hospital, Amiens, France; ^3^INSERM Unit U1105 Research Group for Analysis of the Multimodal Cerebral Function, University of Picardy Jules Verne (UPJV), Amiens, France; ^4^MILA, Paris, France; ^5^CNRS UMR 7222, Institute for Intelligent Systems and Robotics, Sorbonne University, Paris, France

**Keywords:** music therapy, autism spectrum disorder, intellectual disability, neurodevelopmental disorder, systematic review

## Abstract

**Background:** Several studies have reported contradictory results regarding the benefits of music interventions in children and adolescents with neurodevelopmental disorders (NDDs), including autism spectrum disorder (ASD).

**Methods:** We performed a systematic review according to the PRISMA guidelines. We searched the Cochrane, PubMed and Medline databases from January 1970 to September 2020 to review all empirical findings, except case reports, measuring the effect of music therapy on youths with ASD, intellectual disability (ID), communication disorder (CD), developmental coordination disorder (DCD), specific learning disorder, and attention/deficit hyperactivity disorder (ADHD).

**Results:** Thirty-nine studies (*N* = 1,774 participants) were included in this review (ASD: *n* = 22; ID: *n* = 7; CD and dyslexia: *n* = 5; DCD: *n* = 0; ADHD: *n* = 5 studies). Two main music therapies were used: educational music therapy and improvisational music therapy. A positive effect of educational music therapy on patients with ASD was reported in most controlled studies (6/7), particularly in terms of speech production. A positive effect of improvisational music therapy was reported in most controlled studies (6/8), particularly in terms of social functioning. The subgroup of patients with both ASD and ID had a higher response rate. Data are lacking for children with other NDDs, although preliminary evidence appears encouraging for educational music therapy in children with dyslexia.

**Discussion:** Improvisational music therapy in children with NDDs appears relevant for individuals with both ASD and ID. More research should be encouraged to explore whether oral and written language skills may improve after educational music therapy, as preliminary data are encouraging.

## Introduction

The new section of neurodevelopmental disorders (NDDs) in the DSM-5 encompasses psychiatric disorders with an onset in early childhood (1). The clinical expression of all NDDs is closely related to the child's own developmental dynamics. Several mechanisms may explain the structural abnormalities in brain structures reported in most patients with NDDs: consequences of perinatal risk factors, abnormal brain maturation, and consequences of a lack of opportunity to use functional areas. The NDD section of the DSM-5 includes autism spectrum disorder (ASD), intellectual disability (ID), attention deficit disorder with or without hyperactivity (ADHD), developmental coordination disorders (DCD), communication disorders (CD) (including language, phonological, and pragmatic social communication disorders, and stuttering) and specific learning disabilities (SLDs) (characterized by persistent difficulties in learning the fundamental academic skills of reading, writing or mathematics).

### Therapeutic Interventions for ASD

ASD is characterized by two main dimensions: a deficit in communication and reciprocal social interactions and a restriction of interests with repetitive and stereotypical behaviors (1). In addition to the core symptoms of ASD, other developmental dimensions are worth investigating to document associated problems (intellectual functioning, sensory modulation impairments, language difficulties, motor skills, attentional difficulties, emotional regulation, associated medical condition (e.g., seizures), eating, and sleeping) (2). Such integrative approaches help clinicians provide tailored interventions. Three categories of therapeutic interventions for children with ASD have been distinguished:

1. Pure behavioral methods [e.g., *Applied Behavior Analysis* (ABA)] are based on a comprehensive analysis of children's behaviors to promote well-adapted behaviors based on positive reinforcement. Some behavioral methods are based on the child's preferences, such as *pivotal response training* (PRT). Some of these programs involve parents, such as the *Son Rise* program, which occurs at home (3, 4).

2. Developmental methods aim to promote the development process (e.g., *floor time* or the *Early Start Denver Model*—ESDM). In these programs, the therapist starts from the child's interests and follows their initiatives to promote communication performance using the skills of imitation and synchrony. Some of these methods include behavioral techniques, such as ESDM (5, 6).

3. Mixed methods (e.g., *Treatment and Education of Autistic and Communication Handicapped Children*—TEACCH) aim to reduce problematic behaviors in a behavioral approach while considering the child's specific developmental level. Parental guidance is an essential aspect of these programs to allow the child's emerging competences to be generalized into his natural environment (7).

These methods target the primary difficulties experienced by patients with ASD in social interaction and communication, in particular joint attention, imitation, synchrony, emotional sharing and symbolic play. The predictors of the treatment response of patients with ASD to these interventions include intensive interventions (at least 3–4 h per day), interventions provided at an early age, intervention tailored to the patient's developmental needs, interventions promoting family inclusion and spontaneous communication with peers, and interventions with repeated assessment of therapeutic goals based on the child's progress (8).

### Therapeutic Interventions for Other NDDs

Children with ID require a global approach with interventions promoting cognitive skills and autonomy. Most authors recommend that a regular multidimensional evaluation of cognitive, educational, socioemotional, and adaptive skills throughout life. It usually provides a better understanding of how individuals with ID function (9). The goal regarding treatment approach is to contribute toward the planning of more appropriate strategies for learning, care, and support, leading to a better quality of life and participation in society. Specific programs are delivered according to children's developmental levels, family burdens and etiological factors (e.g., antiepileptic drug in case of seizures). Interventions provided at school or specialized institutions play a key role in enhancing pedagogic and academic achievements. Specific intervention form speech therapists, occupational therapists, reading specialists may also target a specific function (9).

For other NDDs, therapeutic approaches directly target the impaired function: speech therapists for children with communication disorders, reading specialists for children with written language difficulties, and occupational therapists for children with motor disorders. Children with ADHD may benefit from attention remediation and cognitive therapy. ADHD is also the only NDD for which effective medications are available (e.g., methylphenidate).

### Music Therapy

From the biblical scene of David playing harp to the *Gnaoua* ritual in Maghreb, anecdotal testimonies of the healing effect of music are reported in all cultures. In the history of psychiatry, music was a component of the moral treatment advocated by Pinel in the eighteenth century. Since music remains one aspect of the milieu and occupational therapy of patients receiving ambulatory care for chronic mental health conditions. It is after the Second World War that music therapy became a structured psychotherapy in North America to treat veteran (10). Nordoff and Robbins (11) created a structured method known as “creative musical therapy” based on the principle of a relational component of music.

Among music therapies, a traditional distinction exists between receptive music therapy techniques (based on listening) and active music therapy techniques (based on sound production by voice, body percussion, or use of instruments). This distinction has been questioned because listening may also involve an active component. Current research usually distinguishes three techniques: music listening, interactive music therapy and improvisational music therapy (12). Interactive music therapy is essentially a structured method including techniques for educational purposes or musical games. For clarity, in this article, the term educational music therapy will be used for this category. Improvisational music therapy uses children's music production to promote spontaneous non-verbal communication. As the distinction between these categories is somewhat arbitrary, a mixed method category also exists.

### Music Therapy for Patients With ASD and/or Other NDDs

The particular interest in music of patients with autism was already noted in the historical description by Kanner (13). He observed that some non-verbal patients are able to sing or hum. Some other patients are able to recognize complex melodies. Several reasons for the hypothesis that music therapy represents a useful adjunct treatment in youths with autism have been documented. Music therapy is regarded as a way of promoting preverbal communication through the improvement of joint attention, motor imitation, and ultimately synchronous rhythm (14). Music therapy has also been used to enhance some cognitive functions, such as attention or memory (15). As impairments in social interactions are also often reported at some point in youths with other NDDs, the effects of music therapy are worth investigating in patients with these other conditions. In this review, we aimed to review the evidence examining the use of music therapy in youths with ASD and/or other NDDs.

## Methods

### Design

The systematic review was conducted according to the recommendations outlined in the PRISMA guidelines (16). Titles and abstracts were scanned for relevance. Full texts were ordered in cases of uncertainty. Reference lists of retrieved systematic reviews were checked. All full texts were assessed for eligibility. Any original study was eligible for inclusion in this review. Abstracts, editorials, and case series with only one evaluation were excluded. Systematic reviews and meta-analyses were examined for references but not included.

We included studies that measured the effect of music therapy intervention (music listening, interactive or educational music therapy, improvisational music therapy, or mixed method), in children or adolescents (participants aged up to 18 years), diagnosed with ASD and/or another NDD. Other NDD were ADHD, ID, DCD, CD, and SLD. Exclusion criteria were: (1) study without outcomes derived from a distinct subgroup of subjects with ASD and/or other NDD; (2) study reporting pooled results without distinction between adults and youths; (3) study that did not provide new empirical data or without objective assessment of clinical outcome; (4) study reporting the effect of music therapy in neurological/neurodevelopmental disorder different from the list mentioned above (e.g., epilepsy).

### Search Method Used to Identify Studies

**Figure 1:**
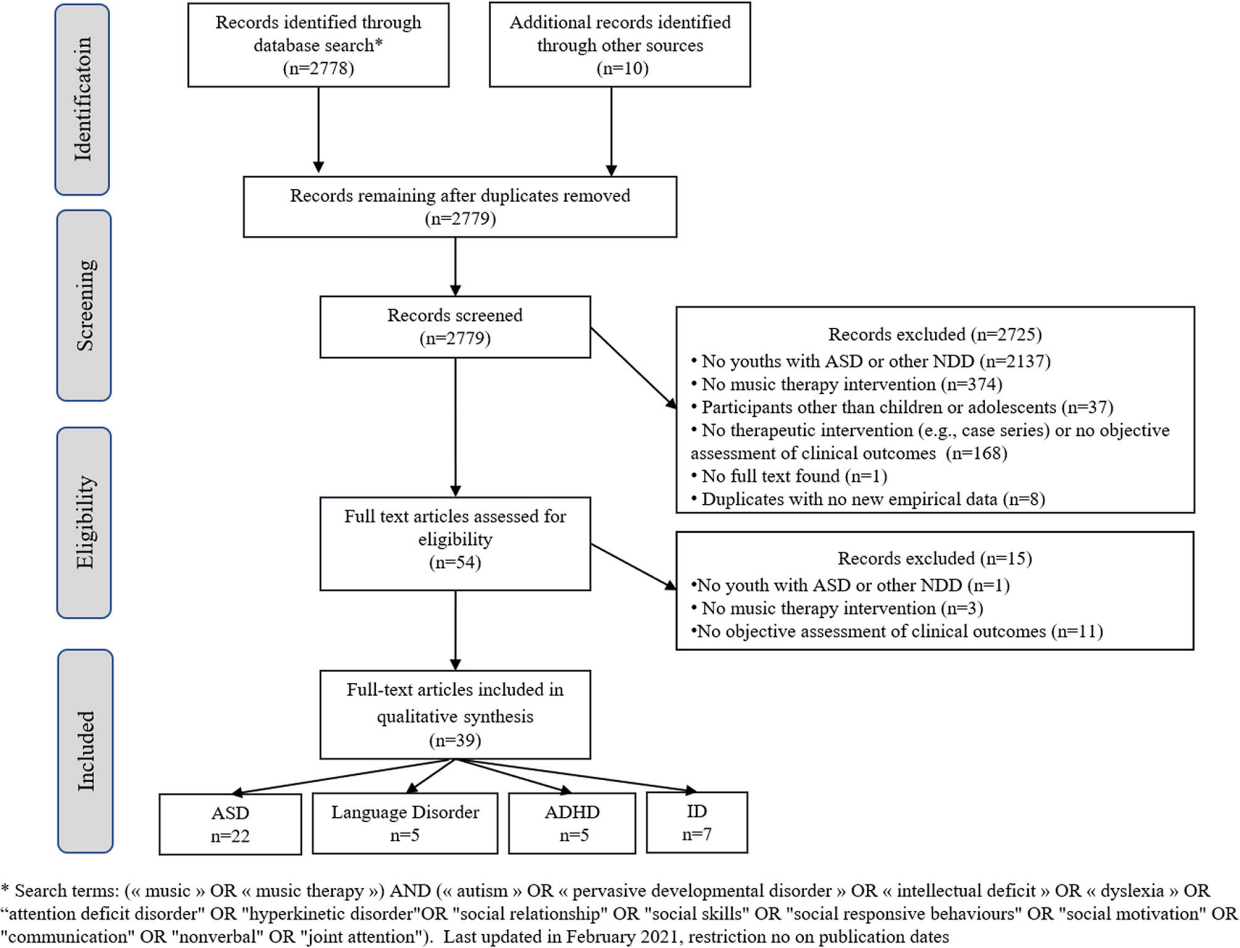
Flowchart of the study.

Relevant articles were obtained by searching the Cochrane Central Register of Controlled Trials (CENTRAL), PubMed and Medline databases. Each database was searched from January 1970 to September 2020. In addition, we hand searched reference lists of identified articles and pertinent reviews for additional studies. Only studies in English, French or German were included. References from the reviewed articles were also screened to find more articles of interest. We used the following search terms: (“*music*” OR “*music therapy*”) AND (“*autism*” OR “*pervasive developmental disorder*” OR “*intellectual disability*” OR “*dyslexia” OR “written language disorder”* OR “*attention deficit disorder*” OR “*hyperkinetic disorder*” OR “*communication disorder*” OR “*oral language disorder*” OR “*specific learning disorder*” OR “*social relationship”* OR “*social skills”* OR “*social responsive behaviours”* OR “*social motivation”* OR “*communication”* OR “*nonverbal”* OR “*joint attention”*). The systematic review yielded 2,778 hits, and 2,725 hits were excluded based on the information in the title or abstract. The full texts of the remaining 54 hits were critically reviewed, leading to the exclusion of another 15 articles because they were only reviews or comments and no new original data were included; alternatively, the research did not present objective outcomes or did not present music therapy intervention or child or adolescent population. One full-text was not found (17). Thirty-nine studies were included: 22 studies of children with ASD, 7 studies of children with ID, 5 studies of children with ADHD, and 5 studies of children with CD and SLD (dyslexia). No study was found for children with DCD, but one ASD study evaluates motor impairment associated with ASD (18). Figure 1 summarizes the PRISMA flowchart of the study.

### Data Analysis

Data and information were independently extracted from each study by the first two authors. In cases of disagreement, a consensus approach was adopted that included the third author. For each study under review, the year of publication and references were extracted. In each report, we collected the following information: (i) description of the sociodemographic and clinical characteristics of the participants (age, gender, and method of diagnosis), (ii) description of the interventions (method of music therapy, duration, frequency, and setting), and (iii) clinical outcomes (effect of the intervention and possible side effects). Considering the disparity in music therapy methods used for children with ASD, the results were presented according to the category of music therapy, i.e., educational music therapy or improvisational music therapy. Only one study included in our review offered a music listening technique, but results were not presented in tables due to severe methodological problems (19). The diverse statistical methods and measurement practices used across studies did not allow for the calculation of pooled effect sizes, such as those used in meta-analyses.

An evaluation of the different risk of bias was performed by the first author according to the Risk Of Bias In Non-randomized Studies—of Interventions (ROBINS-I) tool (20) and the Revised Cochrane risk-of-bias tool for randomized trials (RoB 2) (21). Results are presented in Supplementary Table 1.

## Results

### Music Therapy for Children With ASD

#### Educational Music Therapy (*n* = 272)

Ten studies evaluated the effects of educational music therapy in youths with ASD: five uncontrolled studies (22–26) and seven controlled studies (18, 27–32). They are all summarized in Table 1.

**Table 1:** Studies assessing educational musicotherapy in patients with autism spectrum disorder (ASD).

**Author Year**	**Study design**	**Population**	**Diagnostic**	**Interventions**	**Assessment**	**Main findings**	**Limitations/Comments**
**NON-CONTROLLED STUDIES**							
Lim and Draper (22)	Pre/post evaluation	*N* = 22 Age = 3–5 y.o. Gender: 17 boys Recruitment: na	ASD Dg tool: na ID: na Severity: children were verbal or preverbal with immediate echolalia	**Three different conditions:** Music training: “music incorporated ABA Verbal Behavior”; sung instructions, songs with target words shown on pictures and target phrases with echoic training (A) Speech training: ABA method with the same instructions without singing (B) No specific intervention (C) The order of the three conditions was randomly assigned Settings: individual sessions three times a week for 2 weeks. Duration of each session: na	**Clinical symptom:** no **Interaction:** Observations of behavior in videotaped posttest sessions: Verbal production evaluation scale including semantics, phonology, prosody, and pragmatics **Other assessment:** no	Positive effect on speech production: Verbal production SMD = 9.227 (*p =* 0.000) for MT vs. no training	However, no significant difference in verbal production was observed between music training and speech training The participants scored much higher on echolalia production than in response to questions
Kalas (23)	Pre/post evaluation	*N =* 30 Age = 4–7 y.o. Gender: 28 boys Recruitment: community outpatient activity	ASD Dg tool: na ID: na Severity: CARS: 15 with severe ASD, 15 with mild/moderate ASD	**Two experimental groups:** With simple music condition: simple melody and accompaniment on keyboard With complex music condition: complex melody and complex accompaniment on keyboard (with syncopation or dotted rhythms) Settings: individual 10 min sessions twice a week for 3 weeks	**Clinical symptoms:** no **Interaction:** Behaviors observed in videotaped sessions: ESCS: Joint attention measured by the number of times the child responded to a bid for joint attention by following a pointing gesture toward the object or instrument of interest. RJA score ICC = 0.86 **Other assessment:** no	Statistically significant interaction between the music modality and level of functioning. *F*_(1)_ = 20.089, *p =* 0.001	The effect of simple vs. complex music depended on the level of functioning. Specifically, the simple music condition was more effective at eliciting RJA in children diagnosed with severe ASD, whereas the complex music condition was more effective at eliciting RJA in children diagnosed with mild/moderate ASD
Pasiali et al. (24)	Pre/post evaluation	*N =* 9 Age = 13–20 y.o. Gender: 4 boys Recruitment: special education facilities	ASD Dg tool: na ID: yes Severity: CARS: 3 with severe ASD, 4 with mild/moderate ASD	**Intervention:** Interactive music therapy: “musical attention control training” Settings: 45 min sessions once to twice a week for 6 weeks	**Clinical symptoms:** no **Interaction:** no **Other assessment:** TEA-Ch	Positive changes in scores on tests related to selective attention and attentional control/switching, but no difference in sustained attention	
Paul et al. (26)	Pre/post evaluation	*N =* 3 Age = 3–4 y.o. Gender: 3 boys Recruitment: na	ASD Dg tool: na ID: na Severity: CARS: 1 child with mild to moderate autism, 2 with severe autism	**Interventions** During activities (block matching, picture matching, clay play) (A) Spoken oral instructions (B) Sung oral instructions Settings: individual 15 min sessions once or twice a week, during 3 months. 9 sessions with each condition	**Clinical symptom:** no **Interaction:** Behavior observation of videotaped sessions: performance, frequency of social gesture and eye contact (ICC value correct-to-excellent) **Other assessment:** no	Visual graphic analyze: all participants scored higher in the sung condition compared to spoken condition for all measures	Small sample size No control group
Davis (25)	Pre/post evaluation	*N =* 4 Age = 6–7 y.o. Gender: 4 boys Recruitment: special education facilities	ASD Dg tool: na ID: na Severity: 2 patients (50%) are verbal	**Experimental group:** Interactive music therapy Three different conditions: cooperative music therapy (A), cooperative play (B) and independent play (C) Settings: individual 20 min sessions once or twice a week for 5 weeks	**Clinical symptoms:** no **Interaction:** Behaviors observed in videotaped sessions: joint attention behaviors: interaction (responding to joint attention) and requesting (initiation) behaviors **Other assessment:** no	Interaction: mean difference in scores for MT/independent play = 71 Requesting: mean difference in scores MT/independent play: negative value = −16.875	Increase in interaction behaviors for all subjects during cooperative play and MT compared with independent play, but discordant results for requesting behavior
**CONTROLLED STUDIES**							
Buday (27)	Crossover	*N =* 10 Age = 4–9 y.o. Gender: 8 boys Recruitment: public school	ASD Dg tool: na ID: 70% Severity: CARS: 5 with severe ASD, 5 with mild/moderate ASD	**Two conditions:** (A) Interactive music therapy: songs used to teach signs and speech (B) Rhythmic speech used to teach signs and speech Settings: individual 3 min sessions four times a week for 2 weeks (4 sessions in the first week with one condition and 4 sessions in the second week with the other condition)	**Clinical symptoms:** no **Interaction:** Behaviors observed in videotaped sessions: sign and speech imitating behaviors. ICC 98% **Other assessment:** no	For sign imitation: MT group: M = 5.1 Control group: M = 4.0 Ω^2^ = 0.35/estimated *d* = 0.39 *p <* 0.05 For speech imitation: MT group: M = 4.2 Control group: M = 3.2 Ω^2^ = 0.42/estimated *d* = 0.30 *p <* 0.02	Large effect of music vs. rhythm form on both sign and word learning Limitations: small sample size and only children who have shown an interest in music (obvious attention or enjoyment) were included. Crossover
Farmer (28)	RCT	*N =* 10 Age = 2–5 y.o. Gender: 9 boys Recruitment: na	ASD Dg tool: na ID: na Severity: na	**Experimental group:** Interactive music therapy sessions: guitar playing, songs, games, name and point at body parts, imitation of animals **Control group:** Sessions without music Settings: individual daily 20 min sessions for 5 days	**Clinical symptoms:** no **Interaction:** Behaviors observed in videotaped sessions: Frequency of appropriate verbal and gestural responses **Other assessment:** no	Graphical analysis: Positive effect on verbal responses and gestural responses	Sessions in different conditions (home, school, etc.)
Katagiri (29)	Controlled study	*N =* 12 Age = 9–15 y.o. Gender: na Recruitment: community outpatient activity	ASD Dg tool: na ID: na Severity: na	**Two experimental groups** with background music: prerecorded piano improvisations structured to reference four basic emotions with singing songs: music with lyrics **Active control group**: Verbal instructions Settings: individual 30 min sessions twice a week for 4 weeks **No-intervention control group:** no specific intervention	**Clinical symptoms:** no **Interaction:** no **Other assessment:** Behavioral task: explicit emotional labeling based on facial expression (photographs or schematic drawing) after a learning period	*F* = 2.09, df = 3, *p* = 0.13 Secondary exploratory analysis: Analysis of covariance using pretest scores as a covariate reveals that the background music condition may be the most effective *F* = 8.28 df = 3, *p* = 0.01	None of the four conditions was significantly more effective than the others in improving participants' understanding of the four emotions
Lim (30)	RCT	*N =* 50 Age = 3–5 y.o. Gender: na Recruitment: community outpatient activity	ASD Dg tool: na ID: na Severity: CARS or ADI-R: 25 with moderate/severe ASD, 25 with mild ASD	**Experimental group:** Music training: “Developmental Speech and Language Training through Music”; videotaped songs with target words shown on pictures (PECS) **Active control group**: Speech training: videotaped spoken stories with target words Settings: individual 10 min sessions twice a day for 3 days **No-intervention control group:** no specific intervention	**Clinical symptoms:** no **Interaction:** Behaviors observed in videotaped posttest sessions: Frequency of appropriate verbal responses **Other assessment:** no	Positive effect on speech production: Verbal production (*p <* 0.001) *d* = 1.275 for the MT group vs. no training group	However, no significant difference was observed between the MT group and the speech training group
Sandiford et al. (31)	RCT	*N =* 12 Age = 5–7 y.o. Gender: 11 boys Recruitment: local advertisement	ASD Dg tool: ADOS ID: na Severity: Only children with limited or no functional verbal communication were included	**Experimental group:** Melodic Based Communication Therapy (MBCT): in addition to the traditional word elicitation approach, a combination of listening, hand clapping, singing of the word by the therapist and the child Settings: individual 45 min weekly sessions for 5 weeks **Control group:** Traditional language therapy	**Clinical symptoms:** no **Interaction:** Behaviors observed in videotaped sessions (first and last sessions): number of imitative attempts ICC 96% **Other assessments:** Vocabulary testing based on the International Phonetic Alphabet (assessor is blinded to the intervention): number of verbal attempts and number of correct words Parent survey: number of words reported by the parent	Increase in the number of verbal attempts from weeks 1 through 4 and number of correct words after weeks 1 and 3 in experimental group, while the control group progressed significantly after weeks 4 and 5 No significant differences in the number of verbal attempts (*z* = −1.4, *p =* 0.08) or number of correct words (*z* = −0.2; *p =* 0.05) were observed between the experimental and control groups A significant number of new words were heard in the home environment for the experimental group (z = −2.0, *p* = 0.04), but no significant difference was observed between the two groups (*z* = −0.75; *p =* 0.45) Participants in the experimental group had more imitative attempts (*z* = −2.2; *p* = 0.03)	Small sample size Lack of follow-up: missing data for parent survey
LaGasse (32)	RCT	*N =* 17 Age = 6–9 y.o. Gender: 13 boys Recruitment: local advertisement	ASD Dg tool: na ID: na Severity: CARS2 (values na)	**Experimental group:** Music therapy group: Transformational Design Model, music experiences to facilitate the participants' desired social skills, music-making Settings: 50 min group (3–4 children) sessions, twice a week for 5 weeks **Control group:** Social skills group: cooperative play, including board games and word games. Group interaction included a ball game	**Clinical symptoms:** Parent-rated assessments: SRS (primary outcomes pretest: first group session, posttest: within 4 days of the last group session), ATEC **Interaction: Behaviors observed in videotaped posttest sessions:** eye gaze (ICC 0.934), joint attention (ICC 0.841), initiation of communication (ICC 0.935), response to communication (ICC 0.858), withdrawal behaviors (ICC 0.941) communication (initiation, response and withdrawal) in the first and last sessions **Other assessment:** no	SRS: Significantly greater improvement in the experimental group compared to control group (*p* = 0.032, partial *η^2^* = 0.287) ATEC: Nonsignificant difference between the two groups (*p* = 0.0549) Significant between-group differences in eye gaze toward persons (*p* = 0.022, partial *η2* = 0.323) and joint attention with peers (*p* = 0.031 partial *η2* = 0.291). No significant between-group differences in joint attention with adults, initiation of communication with another child, initiation of communication with an adult, response to communication, or social withdrawal behaviors	Joint attention increased with peers and decreased with adults. Promotion of peer-to-peer interaction Limitations: ATEC may be an inappropriate tool for measuring changes in social skills. Bias due to parental rating Small sample size Higher attrition in the control group. Missing data due to a lack of follow-up
Cibrian et al. (18)	RCT	*N =* 22 Age = 4–8 y.o. Gender: na Recruitment: special education facilities	ASD Dg tool: na ID: developmental age Mean = 5.72, SD = 1.2 Severity: na	**Two experimental groups:** Sound production based on child motor activities (touching, tapping or pinching a spandex fabric with animated background) and different motor exercises using tambourines Settings: individual sessions once a week during 8 weeks, duration of each session na	**Clinical symptom:** Developmental coordination disorder questionnaire **Interaction:** no **Other assessment:** Engagement in music survey: playing in touch questionnaire (clinician-rated) Timing synchronization	Improvements in motor coordination for 27% of participants, and in timing synchronization for all participants	DCD questionnaire may be inappropriate

*y.o., years old; Dg, diagnostic; na, not available; ID, intellectual disability; ICC, Internal Consistency Coefficient; ABA, Applied Behavior Analysis; MT, music therapy; CARS, Children Autism Rating Scale; ESCS, Early Social Communication Scales; RJA, responses to joint attention; TEA-Ch, Test of Everyday Attention for Children; ADI-R, Autism Diagnostic Interview- Revised; PECS, Picture Exchange Communication System; ADOS, Autism Diagnostic Observation Schedule; SRS, Social Responsiveness Scale, completed by guardians; ATEC, Autism Treatment Evaluation Checklist, which was designed to evaluate new treatments through questions on speech and language skills, social skills, physical well-being, and sensory/cognition. The checklist has four areas: speech and communication (14 items), sociability (20 items), sensory/cognitive awareness (18 items), and health/physical behavior (25 items). A lower score indicates higher functioning. The scale was completed by guardians*.

Only one study examined the effect of an educational music therapy intervention on the severity of autistic symptoms (32). The authors observed a small effect of the intervention on the Social Responsiveness Scale (SRS) score (partial η^2^ = 0.29) within the 4 days following the last session. Other studies assessed the effect of educational music therapy on other developmental dimensions. Four studies evaluated the benefit of educational music therapy on joint attention (23, 25, 26, 32). Joint attention was assessed with videos of adult-child interactions by collecting data on pointing behaviors (23, 32), direction of gaze toward an object/person (26, 32), and spontaneous reactions or in response to adults' behaviors (25, 26). None of these studies reported a statistically significant effect of these interventions on the joint attention of children with ASD (23, 25, 26, 32). Paul et al. (26) suggests a positive effect of sung directives during different activities compared to spoken directives on graphic analyzes. Pasiali et al. (24) assessed the effect of music therapy on different components of attention using TEA-Ch (Test of Everyday Attention for Children) in youths with ASD. The authors reported that youths with ASD who received music therapy achieved higher performance in selective and divided attention than at baseline; however, no change in sustained attention was observed before and after the intervention. Another study evaluated the effect of musical therapy on the recognition of emotions in youths with ASD. Katagiri (29) examined whether a piano melody congruent with the emotional valence of a facial expression presented with a picture or a drawing influenced the performance on an emotional recognition task. The authors did not obtain any significant results.

Cibrian et al. (18) evaluated two different music therapy interventions in the motor impairments of young patients with ASD. The reported outcome suggests a positive effect of music therapy on coordination and timing synchronization.

Finally, five studies evaluated the benefits of educational music therapy on language and communication skills (22, 27, 28, 30, 31). In these studies, language was assessed using different methods: list of new words learned (27, 28, 30, 31), spontaneous verbal production (22), and number of words reported by parents (31). Four of five studies showed statistically significant results (22, 27, 28, 30). According to Buday (27), youths with ASD who learned a word list with rhythmic music achieved better results than youths who participated in sessions using the rhythm alone (*d* = 0.30). Farmer (28) observed a tendency in children with ASD who received an intervention with structured musical games to express more appropriate verbal responses than children who received an intervention with non-musical games. Lim (30) showed that the use of music videos facilitated the learning of target words (*d* = 1.28) in children with autism compared to those in a control group that did not receive the intervention. This difference, however, was not statistically significant compared to the group receiving a language intervention based on non-music videos (*d* = 1.14). The same team showed that singing instructions inspired by the ABA method exerted a positive effect on learning target words compared to a control group that did not receive any intervention. This difference was again not statistically significant compared to the group receiving the language intervention (22). Sandiford, Mainess (31) did not report any significant difference in the rate of new words learned between the group of children with ASD who received an intervention using mixed music therapy methods (singing, listening to music and rhythmic hand clapping) compared to children who underwent classical speech therapy sessions. However, an analysis of learning trajectories showed faster progress in the group with music therapy.

#### Improvisational Music Therapy (*n* = 578)

One uncontrolled study (33) and eight controlled studies (14, 34–39, 47) evaluated the effect of improvisational music therapy on youths with ASD (Table 2). Thompson, McFerran (36) measured the effects of home-based improvisational music therapy sessions including family members on the severity of clinical symptoms and the overall level of functioning of youths with ASD. The authors noted that the youths who participated in music therapy sessions had a lower score on the Vineland scale (*d* = 1.96) than the control group who received the current standard of care. No difference was observed in scores on the SRS scale (secondary outcome) between the two groups. Sharda et al. (39) described positive effects of improvisational music therapy sessions compared to games sessions on the level of pragmatic of language (Children's Communication Checklist-2, CCC-2 score) and the quality of life, but no effect on the other main primary outcomes (SRS score and score on the Peabody vocabulary test). Rabeyron et al. (14) documented a higher clinical improvement in the primary outcome (change in Clinical Global Impression, CGI, score, *d* = 0.75) of youths who participated in improvisational music therapy sessions than youths who received an intervention with listening music therapy sessions. The changes observed in the secondary outcomes (i.e., Childhood Autism Rating Scale, CARS, and Autism Behavior Checklist, ABC, scores) were not significantly different between the two groups after the music therapy sessions. Kim et al. (34) observed a positive effect of music therapy sessions on joint attention and prosocial behaviors. Impact of improvisational music therapy on social skills were contradictory: two studies (33, 37) showed an improvement of the Social Skills Improvements System Rating Scale after music therapy sessions, while no significant differences were found in another study (47). Gattino et al. (35) did not find any statistically significant difference in the CARS score between the group receiving music therapy and the group receiving the usual treatment.

**Table 2:** Studies assessing improvisational music therapy in patients with autism spectrum disorder (ASD).

**Authors**	**Methods**	**Population**	**Dg**	**Interventions**	**Assessment**	**Findings**	**Limitations/Comments**
**NON-CONTROLLED STUDIES**							
Yoo and Kim (33)	Pre/post evaluation	*N =* 9 Age = Mean 10.8 y.o. Gender: 8 boys Recruitment: community outpatient activity, special education facilities	ASD Dg tool: na ID: na Severity: CARS: mild to moderate autism Mean = 28.1, SD = 5.7	**Intervention:** Improvisational music therapy, simple rhythmic patterns using dyadic drum playing Settings: 8 individual 30 min sessions. Duration and frequency na	**Clinical symptom:** Parent-rated or teacher-rated assessments: K-SSRS (subscales: cooperation, assertion, and self-control) **Interaction:** Behavior observation of videotaped sessions: occurrence of target behaviors (eye gaze, engagement in joint action, synchronous movements) ICC excellent **Other assessment:** Imitation tasks, asynchrony measures during drum tapping tasks	SSIS: Significant increase in cooperation (*z* = −1.992; *p =* 0.046), self-control (*z* = −2.201; *p =* 0.028) and total score (*z* = −2.201; *p =* 0.028) between pre and posttest No significant improvement in imitation tasks performance, in asynchrony measures Increase in all target behaviors between the first and the last session	No control group Small sample size
**CONTROLLED STUDIES**							
Kim et al. (34)	Crossover	*N =* 10 Age = 3–5 y.o. Gender: 10 boys Recruitment: Child and adolescent psychiatry department in the hospital	ASD Dg tools: DSM-IV TR and ADOS ID: Developmental quotient (PEP) mean 70; range 60–89; SD = 9.97 Severity: 5 children (50%) were non-verbal mean CARS mean = 36.1, range 32–42, SD = 3.41	**Two conditions:** (A) Improvisational music therapy (B) Play sessions with toys Settings: Individual 30 min sessions weekly for 8 months (12 sessions under each condition)	**Clinical symptoms:** no **Interaction:** Behaviors observed in videotaped sessions (mothers and clinicians): - PDDBI rated by mothers and clinicians The ICC between mothers and clinicians was very low (19% pretreatment, 51% between treatments, and 67% posttreatment) The ICC between clinicians ranged from good to excellent - ESCS rated by two independent clinicians: initiation of joint attention (low level: eye contact, high level: pointing) and responding to joint attention bids (number of times the child follows the tester's pointing gesture) The ICC ranged from good to excellent - target behaviors: eye contact duration and turn-taking duration **Other assessment:** no	PDDBI: ANOVA revealed a significant interaction between time and group (*p =* 0.0001) For clinicians' ratings, change during MT vs. change during play: *d* = 0.79 [−014;1.71] ESCS: ANOVA revealed a significant interaction between time and group (*p =* 0.01) Medium effect size in the comparison of scores after MT with scores after play (ignoring sequences): *d* = 0.63 [0.31; 0.95], recalculated based on the change score between data points: *d* = 0.97 [0.21; 1.74]. For the subscales, changes were observed in low initiation of joint attention and responses, but no changes in high level initiation (pointing) Significant effects were observed for eye contact duration (*p <* 0.0001) and turn taking duration (*p <* 0.0001) in the music therapy condition compared with play condition	Overall results generally favored music therapy over the play condition in improving joint attention behaviors Differences in mothers' ratings compared to clinicians': mothers found improvements in both conditions (music and play therapy), whereas clinicians suggested greater improvements after music therapy Marked improvement in joint visual attention skills (eye contact, alternating eye contact with the adult and the object) during and after music therapy compared to play therapy Limitations: small sample size, crossover design, attrition bias
Gattino et al. (35)	RCT	*N =* 24 Age = 7–12 y.o. Gender: 24 boys Recruitment: daily ambulatory care	ASD Dg tools: DSM-IV-TR (autistic disorder, PDD or Asperger syndrome) and ADI-R ID: 30% Severity: CARS-BR mean score = 35.8, range 27–44, SD = 4.4	**Experimental group:** Improvisational music therapy Settings: individual 30 min weekly sessions for 20 weeks **Control group:** no specific intervention	**Clinical symptoms:** CARS-BR: verbal communication, nonverbal communication and social communication **Interaction**: no **Other assessment**: no	Verbal: *p =* 0.50 SMD 0.28, 95% CI [−0.01; 0.57] Nonverbal: *p =* 0.35 SMD 0.39, 95% CI [−0.21; 0.57] Social: *p =* 0.34 SMD 0.39, 95% CI [−0.08; 0.86]	Subgroup analysis: difference across diagnoses (non-verbal communication improved in ASD group but not in PDD or Asperger groups) (*p =* 0.008) Limitations: small sample size, the use of CARS as an assessment may be inappropriate
Thompson et al. (36)	RCT	*N =* 23 Age = 3–6 y.o. Gender: 19 boys Recruitment: community outpatient activity	ASD Dg tool: DSM-IV-TR ID: na Severity: only children with limited or no functional verbal communication were included	**Experimental group:** Songs, improvisation, structured music interactions, in addition to usual care Settings: individual home-based 30 min weekly sessions for 16 weeks including the family **Control group:** Usual care (family-centered early childhood intervention program)	**Clinical symptoms:** Parent-rated assessments: VSEEC (primary outcome), SRS-PS, and MBCDI-W&G **Interactions:** Behaviors observed in videotaped sessions (first and penultimate sessions): MTDA (child engagement in the music therapy sessions) ICC 60% **Other assessments:** PCRI Qualitative data: semi-structured interview of parents	Decrease in the VSEEC score (*p <* 0.001) *d* = 1.96 95% CI [0.92; 3.00] SRS-PS: NS: *p =* 0.341, *d* = 0.42 MBCDI: NS: *p =* 0.553, *d* = 0.26: improvement in both groups, but no significant treatment effect PCRI: NS effect of treatment *p =* 0.099, but *d* = 0.80 MTDA: *p =* 0.001	Significant effect on social engagement (VSEEC and MTDA) Limitations: small sample size Use of parent-reported assessment when parents are not blinded to the intervention
Ghasemtabar et al. (37)	Pre/post evaluation	*N =* 27 Age = 7–12 y.o. Gender: 14 boys Recruitment: community outpatient activity	ASD Dg tool: na ID: na Severity: CARS: mild to moderate Mean = 33.0, SD = 1.9	**Experimental group:** Improvisational music therapy: music hearing, singing songs, hand clapping, dancing, free and creative playing of instruments Settings: group 1 h sessions, twice a week during 6 weeks **Control group:** Usual care	**Clinical symptom:** Parent-rated assessments: SSRS (sub-scales of cooperation, assertions, self-control and responsibility) **Interaction:** no **Other assessment**: no	Significant higher improvement in SSRS score in posttest in the experimental group compared to controls, however no significant difference was observed at follow-up (2 months after the last session)	Small sample size Use of parent-reported assessment whereas parents are not blind to the intervention Non-cooperative children were excluded, attrition bias
Porter et al. (47)	Subgroup analysis of RCT	*N =* 34 Age = 8–16 y.o. Gender: na Recruitment: community outpatient activity	ASD-NS Dg tool: na ID: na Severity: na	**Experimental group:** Improvisational music therapy: Voice, movement and instrument playing Settings: individual 30 min weekly sessions during 12 weeks **Control group:** Usual care	**Clinical symptom:** Parent-rated assessments: SSIS (primary outcome) Social functioning subscale of the CBCL, CES-D **Interaction:** no **Other assessment:** Rosenberg self-esteem scale Family functioning scale	Non-significant difference between experimental and control groups with regards to SSIS total score at week 13 (mean difference 3.6 [95%: −4.6;12.0], *p =* 0.37)	Subgroup analysis
Bieleninik et al. (38)	RCT	*N =* 364 Age = 4–6 y.o. Gender: 308 boys Recruitment: community outpatient activity	ASD Dg tools: CIM10, ADOS, and ADI-R ID: 46% Severity: ADOS (mean value at baseline = 17.7)	**Experimental groups:** Improvisational musicotherapy Settings: individual 30 min sessions, possibly joined by family members for 5 months, with two different frequencies: High intensity MT: 3 times a week Low intensity MT: once a week **Control group:** Usual care Follow up: 1 y	**Clinical symptoms:** ADOS SRS (parent-rated) **Interaction:** no **Other assessment**: no	Primary outcome: ADOS (5 M) MD 0.06 [95%: −0.70;0.81] *p =* 0.88 Significant results obtained for several secondary outcomes: Greater improvement in SRS-social awareness for the low-intensity MT group compared with the control group at 2 months (*p =* 0.004) Greater improvement in SRS-social motivation scores for the MT (low+high intensity) than in the control group at 12 months (*p =* 0.007) Greater improvement in SRS-autistic mannerisms scores for the high intensity MT group compared with the control group at 5 months (*p =* 0.009)	Large RCT and encouraging secondary analysis (see discussion) Limitations: Observed differences in scores on social responsiveness subscales may be artifacts attributable to multiplicity and lack of blinding
Sharda et al. (39)	RCT	*N =* 51 Age = 6–12 y.o. Gender: 33 boys Recruitment: community outpatient activity	ASD Dg tool: DSM IV-TR, ADOS, ADI-R or CARS ID: no (WASI-II full-scale scores M 100, SD 15) Severity: ADOS (mean ~15)	**Experimental group:** Improvisational music therapy approaches: musical instruments, songs and rhythmic cues while targeting communication, turn-taking, sensorimotor integration, social appropriateness and musical interaction Settings: individual 45 min sessions weekly for 8 to 12 weeks **Control group:** Play-based intervention	**Clinical symptoms:** Parent-rated assessments: CCC-2, SRS-II, PPVT-4, FQoL and the maladaptive behaviors subdomain of the VABS (VABS-MB) Language ability assessments: CELF-4 and PPVT-4 **Interaction: no** **Other assessment:** Functional neuroimaging: resting-state connectivity of frontotemporal brain networks	Primary outcomes: “social communication battery”: CCC-2, SRS-II and PPVT-4: Increase in the communication score of the CCC-2 in the experimental group after the intervention compared to the control group (*p* = 0.01) *d* = 0.34 Improvements were specific to pragmatics, reduction of inappropriate initiations and better social relations and interests. No significant results were obtained for SRS-II or PPVT-4 Secondary outcomes: FQol and VABS-MB Significant effect of music therapy on FQoL (*p =* 0.01, *d* = 0.57) compared to the control group. Both groups showed a reduction in the VABS-MB score (post-intervention *p =* 0.01) Post-intervention resting-state brain functional connectivity was i) greater between auditory and subcortical regions and auditory and fronto-motor regions and ii) lower between auditory and visual regions in the music compared to the non-music groups	Improvement only in the CCC-2 score, which measures pragmatic communication. MT may have exerted a limited effect on the ASD symptom severity or on improving receptive vocabulary Music employs a structured approach similar to social communication, which may otherwise be hindered by sensory and social difficulties Changes in brain connectivity were related to improvements in children's communication skills after MT. Music might play a modulatory role in reducing the overconnectivity between sensory cortices, subsequently improving communication processes Limitations: small sample size
Rabeyron et al. (14)	RCT	*N =* 36 Age = 4–7 y.o. Gender: 31 boys Recruitment: na	ASD Dg tool: na ID: na Severity: CARS, ABC	**Two experimental groups:** Improvisational music therapy Music listening Settings: group (3–5 children) 30 min sessions, weekly for 8 months	**Clinical symptoms:** CGI, CARS, and ABC **Interaction:** no **Other assessment:** Qualitative patient-centered analysis	Primary outcome: CGI: *d* (estimated) = 0.75; *p =* 0.028 Secondary outcomes: CARS, ABC: no effect	Blind assessment of the primary outcome Limitations: small sample size

*y.o., years old; dg, diagnostic; na, not available; ADOS, Autism Diagnostic Observation Schedule; ID, intellectual disability; SSIS, Social Skills Improvement System rating scale; CBCL, Child Behaviour checklist; CES-D, Center for Epidemiological studies depression; PEP, Psycho-Educational Profile; CARS, Childhood Autism Rating Scale; PDDBI, Pervasive Developmental Disorder Behavior Inventory; ESCS, Early Social Communication Scale; MT, music therapy; PDD, pervasive developmental disorder; ADI-R, Autism Diagnostic Interview-Revised; CARS-BR, Childhood Autism Rating Scale adapted for Brazil: 3 measured outcomes: verbal communication, non-verbal communication and social communication; CARS2, Childhood Autism Rating Scale- second edition; ATEC, Autism Treatment Evaluation Checklist, which was designed to evaluate new treatments through questions on speech and language skills, social skills, physical well-being, and sensory/cognition. The checklist has four areas: speech and communication (14 items), sociability (20 items), sensory/cognitive awareness (18 items), and health/physical behavior (25 items). A lower score indicates higher functioning. The scale was completed by guardians. SRS, Social Responsiveness Scale: completed by guardians; SRS-PS, Social Responsiveness Scale, preschool version for 3 y.o. It assesses impairments independent of the child’s IQ in repetitive behaviors, interpersonal behavior and communication. VSEEC, Vineland Social Emotional Early Childhood scale. The parent assessed the quality of the child's interactions in the home and community according to how well the child gives attention, enters into intentional social interactions, and understands and expresses emotion. Only two of the three subscales were used in the study by Thompson et al. (36): interpersonal relationship and play/leisure time. MBCDI-W&G, MacArthur-Bates Communicative Development Inventories, Words & Gestures: assessment of early language skills in the areas of vocabulary comprehension and production, and use of communicative gestures; PCRI, The Parent–Child Relationship Inventory: assesses parent attitude toward parenting and their child; MTDA, Music Therapy Diagnostic Assessment: child engagement in the music therapy sessions; ABC, Abberant Behavior Checklist; CCC-2, Children's Communication Checklist; VABS-MB, maladaptive behavior subscale of the Vineland Adaptive Behavior Scales; SRS-II, Social Responsiveness Scale; FQoL, Beach family Quality of Life scale; WASI-II, Wechsler's Abbreviated Intelligence Scale; CELF-4, Clinical Evaluation of Language Fundamentals; PPVT-4, Peabody Picture Vocabulary Test; CGI, Clinical Global Impression, K-SSRS, Korean-Social Skills Rating System*.

The study by Bieleninik et al. (38) deserves more attention, as the study included 364 young patients with ASD in 9 different country sites. They did not observe a significant difference in the Autism Diagnostic Observation Schedule (ADOS) or SRS score (primary outcomes) at 2, 5, and 12 months between the three arms: treatment as usual, non-intensive music therapy, and intensive music therapy. However, they identified some differences in scores for SRS subscales, with a greater improvement in the score for the social motivation subscale of the music therapy group at 12 months and in the score for the autistic mannerisms subscale at 5 months. In addition, the authors presented at a congress unpublished data stressing that the subgroups of subjects with both ASD and ID had a higher response rate for the primary outcome ADOS (risk ratio = 1.43 [95% CI: 1–2.05], *p* = 0.049).

### Music Therapy for Children With Other NDDs

#### Intellectual Disability (*n* = 361)

Five non-controlled studies (40–44) and two controlled studies (45, 46) evaluated the effect of music therapy on youths with ID (Table 3). Two studies showed a positive effect of music therapy sessions on parent-child interactions, with increased spontaneous demands by the child and adapted parental responses (41, 43), more synchronous behaviors between parents and children (43), and an improvement in parents' mental health (41). Zyga et al. (44) also reported an improvement in the socioemotional abilities of children who participated in mixed music therapy sessions, including singing, dancing and theater. Mendelson et al. (42) documented a positive effect of educational music therapy sessions delivered in the classroom on peer interactions during the sessions. However, this effect was observed only in subjects who participated in a 15 week program and not in participants who received a shorter 7 week intervention.

**Table 3:** Studies assessing musicotherapy in youths with intellectual disability (ID).

**Authors**	**Methods**	**Population**	**Dg**	**Intervention**	**Assessments**	**Findings**	**Limitations/Comments**
**NON-CONTROLLED STUDIES**							
Rainey Perry (40)	Qualitative case study	*N =* 10 Age = 5–12 y.o. Gender: 8 boys Recruitment: na	ID Severe and multiple disabilities; mostly sensory impairment and neurological disorders	**Intervention:** improvisational music therapy Settings: 5 individual sessions. Duration of each session and frequency: na	Analysis of videotaped sessions: rating of a “communication profile.” Describe turn taking, type of improvisation and interactions (musical and non-musical)	Descriptions of interactions in one chosen videotaped session for each child	This study suggests that children may need the possibility of responding to an interaction at their level, and may benefit from being encouraged in their engagement in communication: singing or playing together and not only taking turns, similar to early preverbal communication
Williams et al. (41)	Pre/post evaluation	*N =* 201 Age = 3–60 months Gender: na Recruitment: community outpatient activity	Global developmental delay (32%), ASD (15%), speech and language impairments (18%)	**Intervention:** singing songs with movements, playing instruments, quiet music to encourage parent-child bonding Settings: group (8–10 parent-child pairs) 60 min sessions once a week for 10 weeks	Parent-reported assessments: Parental mental health symptoms (Kessler K6, auto questionnaire) Parenting self-efficacy Parent-child interactions: parental responsiveness/warmth (child rearing questionnaire), irritable parenting (parental perceptions and behaviors scale), parental engagement in home learning activities with their child Child behaviors (mood and behavior subscale of the NEILS scales of developmental competency) Social play skills, receptive communication skills (subscale of the NEILS scales of developmental competency) Social support, satisfaction and perceived benefits Clinician assessments: 6-item observational checklist for the first two and last two sessions evaluating quality of parental behavior toward the child and child behavior toward the parent and others. A second observer independently coded behavior. The ICC was excellent	Symptoms Improvement in parents' mental health, child's communication, and child's social play No change in child's behavior problems, activities with child, parenting irritability, self-efficacy and warmth Behaviors Improvements in all measures (parent sensitivity, parent engagement, parent acceptance, child responsiveness, child interest, child social engagement)	
Mendelson et al. (42)	Pre/post evaluation	*N =* 33 Age = na, 2nd graders Gender: na Recruitment: special education facilities	ID 5 children with ASD, 32 without ASD Severity: na	**Intervention**: VOICSS method (vocal interactive communication and social strategies): interactive music therapy using songs with a high expectation of a reciprocal response, turn taking Settings: group, classroom-based 45 min sessions once a week during 7 weeks (short-term music therapy) or 15 weeks (long term music therapy)	Teachers' ratings: Social Skills Improvement System-Rating Scale (SSIS-RS) Behavioral observations: child's verbal and social responses coded on a Likert scale by two raters during live observations in the classroom. The ICC was fair to excellent	SSIS-RS: no significant difference During the therapy sessions, levels of social and communicative responses showed significant differences in the long-term program, but not in the short-term program	Probable dose-effect
Yang (43)	Pre/post evaluation	*N =* 26 (parent-child pairs) Age = 1–3 y.o. Gender: 20 boys Recruitment: community outpatient activity	ASD (46%) ID (23%) Language delay (19%) Severity: na	**Intervention:** interactive music therapy (singing songs together, musical games) with the child and his parent Settings: individual home-based 40 min sessions once a week for 6 weeks	Analysis of videotaped sessions: - parent-child interaction (children initiations and parents' response during free-play sessions) coded by two independent raters. The ICC was excellent - parent-child synchrony (same focus of attention, matched affect and reciprocal and responsive exchanges): Likert scale. The ICC was excellent	Significant improvements between pre and posttest scores for parents' physical and verbal responses, for children's verbal initiation and for parent-child synchrony No significant difference in children's physical initiation	
Zyga et al. (44)	Pre/post evaluation	*N =* 47 Age = na, 1st grade to 12th grade Gender: na Recruitment: special education facilities	ID ASD, attention disorder, specific learning disorder Severity: na	**Intervention:** Kids Love Music program: learning the story, choreography and songs from The Wizard of Oz Settings: in-school 30–45 min group sessions (5–12 children) twice a week for 4 weeks	Analysis of videotaped sessions (first and last sessions): socioemotional skill scale: eye contact, turn taking, engagement, social awareness, symbolic flexibility, and emotional understanding	Significant changes in all domains	Missing data on population Not controlled results
**CONTROLLED STUDIES**							
Aldridge et al. (45)	Crossover	*N =* 12 Age = 4–6.5 y.o. Gender: 3 boys Recruitment: community outpatient activity	ID Severity: na	**Two conditions:** (A) Improvisational music therapy Settings: individual 30 min sessions once a week for 2 weeks. (B) Waiting list	Griffiths tests (locomotor developmental, personal-social hearing and speech, hand-eye coordination, performance, and practical reasoning)	Significant change during the first study period; when the Waiting List Group was treated and then tested at Test 2, the newly treated children started to catch up in their development	The activity of listening in a structured musical improvisational context without the lexical demands of language is a platform for improving communication Hand-eye coordination, which depends on a wider body awareness, appears to be a vital component of developmental changes
Duffy and Fuller (46)	Case control study	*N =* 32 Age = 5–10 y.o. Gender: na Recruitment: special education facilities	ID Severity: moderate	**Experimental group:** group music therapy social skills program using prerecorded music **Control group**: non-music group Settings: 30 min group sessions twice a week for 8 weeks	Likert scale evaluating social skills, based on the analysis of the first and the last videotaped sessions - social skill measure specifically developed for the study including turn-taking, imitation, vocalization, initiation and eye contact - researcher and independent observer	Significant increase in all dimensions, but no difference between the two groups. The music therapy appeared to show a tendency toward being more effective only one skill area (i.e., imitation)	

*y.o., years old; dg, diagnostic; na, not available; ASD, autism spectrum disorder*.

Regarding controlled studies, Aldridge et al. (45) showed a non-significant trend for a positive effect of improvisational music therapy sessions on a global measure of the developmental level in children with ID. Specifically, the authors stressed the importance of the improvement in hand-eye coordination. Duffy and Fuller (46) did not detect a significant difference in the rate of progress in terms of imitation, vocalization, initiation of interaction, eye contact and turn-taking between children who received music therapy and those who did not.

#### Attention Deficit Hyperactivity Disorder (*n* = 443)

Three non-controlled studies (48–50) and two controlled study (51, 52) evaluated the effect of music therapy on youths with ADHD (Table 4). These studies presented significant methodological biases. The largest study (*n* = 268) was based on a questionnaire administered to music therapists (34). Rothmann et al. (52) showed significant improvements in attentional performance tests and in quality of life after music therapy among children with suspected ADHD compared to those who received usual care. Montello and Coons (48) suggested that school-based educational music therapy sessions using rhythms are associated with increased attention and motivation and decreased hostility in children with behavioral problems based on teacher reports.

**Table 4:** Studies assessing musicotherapy in youths with attention deficit hyperactivity disorder (ADHD).

**Authors**	**Methods**	**Population**	**Dg**	**Interventions**	**Assessments**	**Findings**	**Limitations/Comments**
**NON-CONTROLLED STUDIES**							
Montello and Coons (48)	Pre/post evaluation	*N =* 16 Age = 11–14 y.o. Gender: 14 boys Recruitment: special education facilities	“emotional disturbance,” learning disabilities and/or attention deficit disorder Dg tool: na ID: na Severity: na	**Two interventions:** “Active group”: rhythm-based intervention “Passive group”: listening-based intervention **Three groups:** A: active and passive B: passive and passive C: passive and active Settings: group 45 min sessions once a week for 12 weeks	Teacher's interview (Achenbach) evaluating attention, motivation and hostility	Improvements were observed in the groups using the rhythm-based intervention	
Jackson (49)	Review of clinical practice	268 questionnaires completed by music therapists	ADHD Dg tool: na ID: na Severity: na	Several music therapy interventions	Perceived effectiveness of musicotherapy on ADHD	Most of the therapists describe music therapy as effective in children with ADHD	
Gooding (50)	Pre/post evaluation	*N =* 45 Age = 6–17 y.o. Gender: na Recruitment: public school	ADHD, dyslexia, specific learning disabilities and/or Asperger, PTSD, anxiety disorder Dg tool: na ID: no Severity: na	Music therapy-based social skills intervention program at school, home and after school care settings 50 min weekly group sessions for 5 weeks	Likert-type ratings of participants by teachers, researchers and the participants themselves evaluating social functioning after the first and fifth sessions. Appropriate communication behaviors during the observation period	Non-significant results	Substantial heterogeneity in participants and intervention settings Not randomized
**CONTROLLED STUDIES**							
Rickson (51)	Case control study	*N =* 13 Age = 11–16 Gender: 13 boys Recruitment: special education facilities	ADHD Dg tool: DSM-IV ID: most have mild ID Severity: treated with stimulants	**Two experimental groups:** - Instructional intervention: structured rhythmic exercises, positive reinforcement - Improvisational intervention **Control group:** waiting list	Conner's rating scale for parents and teachers Synchronized tapping tasks	No significant results	Not randomized
Rothmann et al. (52)	Case control study	*N =* 101 Age = 5–10 y.o. Gender: 75 boys Recruitment: community outpatient activity	ADHD or suspicion of attention difficulties Dg tool: na ID: na Severity: naïve of medication	**Experimental group:** Educational music therapy program involving rhythm exercises with percussion instruments and musical games Settings: Group (4–6 children) 1 h session once a week during 18 weeks **Control group:** Usual care	Test of attentional performance for children Quality of life ratings Symptom checklist for ADHD Symptom checklist for conduct disorder	Significant improvement in attention performance (*p <* 0.0001) and in quality of life (*p <* 0.0001) in the experimental group compared to control group	No formal diagnostic of ADHD No active control group

*y.o., years old; dg, diagnostic; na, not available*.

#### Communication Disorders and Specific Learning Disabilities (Dyslexia) (*n* = 120)

Four studies were conducted in youths with dyslexia, and one study was conducted in youths with language delay (Table 5). Two non-controlled studies examined the effect of educational music therapy on children with dyslexia (53, 54). Overy (53) reported a positive effect of school-based music therapy sessions on reading competence in nine children with dyslexia. Habib et al. (54) documented an improvement in several domains of written language competence in 12 children with dyslexia after music therapy sessions, such as phonological perception, pseudoword repetition, word reading, and auditory attention.

**Table 5:** Studies assessing musicotherapy in children with communication/oral and written language disorder.

**Authors**	**Methods**	**Population**	**Dg**	**Interventions**	**Assessments**	**Findings**	**Limitations/Comments**
**NON-CONTROLLED STUDIES**							
Overy (53)	Pre/post evaluation	*N =* 9 Age = mean 8,8 y.o. Gender: 9 boys Recruitment: public school	Dyslexia Dg tool: dyslexia screening test ID: na Severity: na	**Intervention**: instructional program with singing songs Settings: school-based 20 min group lessons three times a week for 15 weeks	Language and literacy tests	Significant positive effect of the music program on rhythm copying, rapid auditory processing, phonological ability, and spelling ability	
Groβ (57)	Pre/post evaluation	*N =* 18 Age = 3.5–6 y.o. Gender :12 boys Recruitment: public school	Delayed speech (excluding ASD and/or muteness or speech developmental disorder) Dg tool: na ID: na	**Intervention:** improvisational music therapy (Nordoff and Robbin's method): singing songs, percussion instruments and piano playing Settings: individual 25 min sessions. Frequency and number of sessions: not available	- Standardized speech development test: understanding of sentences, speech production, and memory of speech - Nonverbal developmental test - Analysis of videotaped sessions: child-therapist relationship in musical activity and “musical communicative activity.” ICC: 82%	Positive trends for phonological memory and understanding of sentences Better relationship between child and therapist over time Significant decrease in the difference between developmental and chronological ages	
Habib et al. (54)	Pre/post evaluation	*N =* 12 Age = 7–12 y.o. Gender: 8 boys Recruitment : special education facilities	Dyslexia Dg tool: na ID: na Severity: severe dyslexia, mean difference between chronological age and reading age at 36 months	**Intervention:** “Cognitive-Musical Training”: transmodal intervention, use of rhythm with body parts and piano keyboard. Structured intervention Settings: Two 30 min group sessions (group of 4 children with the same level) and 2 1 h group sessions all 12 children per week for 6 weeks	Several tasks selected from the NEPSY II battery, which evaluate categorical perception of syllables, attentional processing and phonological, reading tasks, visual and writing tasks	After the intervention, improved categorical perception of syllables, auditory attention, pseudowords repetition, reading words, phonological awareness and comparison of letter strings	Methodology tested on a pilot study including 12 subjects receiving 3 days of musical training
**CONTROLLED STUDIES**							
Register et al. (55)	RCT	*N =* 33 (8 students with specific disability in reading) Age = na, 2nd graders Gender: na Recruitment: public school	Dyslexia Dg tool: na ID: na Severity : na	**Experimental group:** instructional program with singing songs **Control group:** traditional program Settings: group school-based sessions three times a week for 4 weeks. Duration of each session: na	Vocabulary and reading comprehension tests	For students with a specific disability in reading, the musical program may effectively improve reading comprehension	
Flaugnacco et al. (56)	RCT	*N =* 48 Age = 8–11 y.o. Gender: 34 boys Recruitment : community outpatient activity	Dyslexia Dg tools: anamnestic interview and neuropsychological assessment ID : no Severity: na	**Experimental group**: music training: use rhythm (percussive instruments and rhythmic body movement) and sensorimotor synchronization games **Control group:** painting training Settings: 1 h group sessions (5–6 children) twice a week for 30 weeks (excluding holidays)	**Symptoms:** - phonological awareness (pseudoword repetition test of the Promea Battery): phonemic blending and phonemic segmentation - reading abilities: reading a text aloud (MT reading test), DDE-2: reading single words and pseudowords (primary outcome) aloud **Other assessments:** Working memory (WISC III) and self-esteem (multidimensional test of self-esteem-TMA)	Improvements in several reading tasks in both groups (no difference in reading speed between two groups), better improvement in the experimental group compared to control group in: - text reading: 50% fewer poor performers in the experimental group compared to the control group after the intervention - accuracy in reading pseudowords - phonological abilities - working memory - auditory attention The outcome in the rhythm production task is a predictor of phonological awareness (phonemic blending and phonemic segmentation). A greater improvement in rhythmic abilities indicates a greater improvement in phonological awareness (bending task) (*p =* 0.002)	Supports the hypothesis of a causal role for rhythm-based processing in language acquisition and phonological development Recommends an interest in the use of music as a complementary tool in reeducation

*y.o., years old; dg, diagnostic; ID, intellectual disability; NEPSY, a developmental NEuroPSYchological Assessment; DDE-2, Assessment Battery for Developmental Dyslexia and Dysorthography, second version; MT, music therapy; WISC III, Wechsler's Intelligence Scale for Children, third edition; TMA, multidimensional self-esteem test: the Italian version of the Multidimensional Self-Concept Scale*.

Two randomized controlled studies were conducted to document the effect of educational music therapy on children with dyslexia. Register et al. (55) observed an improvement in vocabulary and reading comprehension test scores in children with written language difficulties who participated in school-based music therapy sessions compared to children who participated in a traditional program for learning difficulties. Flaugnacco et al. (56) found that children with dyslexia who participated in school-based music therapy group sessions presented increased phonological awareness and reading skills (for accuracy but not speed) compared to children who had participated painting sessions during the same time. The authors noted that rhythm perception during the sessions was a predictor of a positive treatment response.

Finally, one non-controlled study (57) evaluated the effect of improvisational music therapy on 18 children with language delay. The authors observed a significant increase in developmental age (in particular phonological memory and sentence understanding) after a 6 week individual improvisational music therapy program.

## Discussion

### Main Findings

Considering the diversity of music therapy approaches, improvisational and educational music therapy programs provided to youths with ASD and other NDDs were distinguished. Regarding educational music therapy, our findings support a positive but small effect of educational music therapy on children with NDDs, particularly patients with ASD and/or ID. Two major limitations in the results obtained for children with ASD are noted. First, only one study used core ASD symptoms as a primary outcome (32), leading to difficulties in generalizing the results. Second, the results for improving joint attention are mixed. The determination of whether educational music therapy interventions are ineffective or whether these effects are difficult to demonstrate is challenging. Indeed, joint attention measures are rarely standardized, questioning their validity.

Studies evaluating the effect of educational music therapy on language and communication skills in children with ASD reported three main results. First, a significant effect on learning target words based on imitation skills was observed. Second, a study showed that educational music therapy sessions were associated with improvements in several components of oral language (phonology, semantics, prosody, and pragmatics) (30). Third, a positive effect was observed for the music therapy group compared to the group without treatment, but not for the active control group using non-musical techniques.

Regarding improvisational music therapy, we found few empirical findings supporting a positive effect of improvisational music therapy sessions on youths with NDD, but some findings appear interesting for children with ASD and/or ID. The most methodologically robust study conducted by Bieleninik, Geretsegger (38) did not report a significant improvement in the primary outcome (ADOS) and a positive effect of music therapy sessions only on a few secondary outcomes. However, they observed a significant effect on the subgroup of participants with ASD and ID. In a secondary analysis based on what aspects of improvisational music therapy predicted an improvement, the authors performed a microanalysis of the video therapy sessions and showed that a high level of relational adjustment between the child and the therapist was a strong predictor of positive outcomes (58). These authors concluded that the intervention is more effective when the therapist adopts a relational pattern similar to the child's pattern. A similar hypothesis was formulated by Rainey Perry (40) based on their work with children with severe and multiple disabilities. The therapist uses a relational mode that fits the child's “communication profile” to provide the child opportunities to respond to interactions.

The choice of the primary outcomes in the reviewed studies should be discussed. In the study conducted by Bieleninik, Geretsegger (38), the primary outcome was the social affect score of the ADOS at 5 months (i.e., encompassing problematic reciprocal social interactions and communication). However, the ADOS is a diagnostic tool that was not specifically designed to assess variations in clinical severity over time. However, the studies that used clinical scales such as the SRS or the CARS to track changes in the severity of autistic symptoms did not present significant findings. However, several reviewed studies showed a positive effect of improvisational music therapy sessions on children with ASD or/and ID using measures of subjective clinical improvement (e.g., CGI) and the level of global functioning or quality of life (14, 36, 39). Finally, music interventions including family members exerted a stronger effect than other types of interventions for youths with ID (36). This finding is a possible argument supporting the inclusion of family in improvisational music intervention sessions in youths with ID. However, so far, no direct comparison between therapy sessions including family members or not has been conducted to test this hypothesis.

Regarding children with NDDs other than ASD and/or ID, two observations can be made from the preliminary data on the effect of musicotherapy. First, a positive trend for music therapy sessions using rhythm on the written language of children with dyslexia was reported. However, the number of studies and the total number of children included (*n* = 120) remain limited. Second, the quality of evidence supporting a positive effect of music therapy on children with ADHD remains poor.

### Limitations

Currently, many limitations exist regarding the studies included in the current review. The main limitation of this review is related to the methodological quality of the studies analyzed, with small sample sizes and wide age ranges (Supplementary Table 1). Most studies had a non-controlled design, and when a control group existed the allocation of the treatment was not necessarily randomized (e.g., case-control study) questioning the impact of possible confounding biases. Second, music therapy interventions are extremely heterogeneous, particularly in the context of educational music therapy, making cross-study comparisons difficult and meta-analysis calculations invalid. Third, the primary outcomes used varied widely between studies. A lack of information about the measures of interaction, if any, contributes to the heterogeneity of the studies. Associated evaluations of clinical dimensions have rarely been performed. In particular, no study included a standardized assessment of anxiety symptoms, while previous reports show a benefit of music therapy sessions in patients with these symptoms (59). Finally, the conclusion of this review may be influenced by the process of study selection and the limitations inherent to the search strategy (e.g., lacking of keywords, studies in other language, publication bias).

### Implications

How useful is the addition of music therapy sessions to the traditional care of children with NDDs? A number of methodological biases prevents us from generating any firm conclusions based on the studies reviewed. Among the different combinations of music therapy sessions and clinical characteristics of the patients tested, a stronger effect was observed for the use of improvisational music therapy in children with both ASD and ID (22, 24, 31). While these interventions failed to significantly reduce the level of autistic symptoms in most published studies, several authors reported a positive effect on other clinical dimensions (22), on the overall level of functioning (e.g., CGI) (24), and on quality of life (39, 41). Notably, the effect was enhanced when family members were included in sessions (36, 41, 43).

Further studies would be particularly inspired to document the potential mediators of the therapeutic effects (e.g., age of the participants; cognitive characteristics), in addition to the measurements of clinical symptoms and level of functioning (58). Among other hypotheses, music therapy sessions might exert a positive effect by increasing the quantity and quality of adult-child interactions. The child-therapist relation might be regarded as an “experimental” relation for children, where he/she learns to attune his/her behaviors to adult behaviors. Another promising finding that deserves attention is the positive effect of educational music therapy on children with dyslexia, but more research is needed to conclude any definitive positive effect. For children with other NDDs, more substantial studies are needed before a conclusion can be made on the value of their use.

## Author Contributions

DC and HM-B designed the study and wrote the first draft of the manuscript. HM-B, XB, and FV performed the study search. HM-B and XB extracted the data from the studies. FV checked the data for consensus agreement. All authors contributed to the final version of the manuscript.

## Conflict of Interest

The authors declare that the research was conducted in the absence of any commercial or financial relationships that could be construed as a potential conflict of interest.
