# Supplementary material for: Music Therapy for Children With Autistic Spectrum Disorder and/or Other Neurodevelopmental Disorders: A Systematic Review
Source: Front Psychiatry. 2021 Apr 9;12:643234. doi: 10.3389/fpsyt.2021.643234 (PMC8062803; doi:10.3389/fpsyt.2021.643234)
Supplement: Supplementary file 1 [file Data_Sheet_1.docx]

**Table S1.** Risk of bias in non-randomized studies using the ROBINS-I scale

| **Authors** | **Bias due to confounding** | **Bias in selection of participants into the study** | **Bias in classification of interventions** | **Bias due to deviations from the intended intervention** | **Bias in measurement of outcomes** | **Bias to missing data** | **Bias in selection of the reported result** | **Overall bias** |
| --- | --- | --- | --- | --- | --- | --- | --- | --- |
| **ASD** | | | | | | | | |
| Buday 1995 | H | SC | SC | H | L | L | L | SC |
| Kim 2008 | SC | L | SC | H | H | H | L | SC |
| Katagiri 2009 | SC | H | L | H | H | L | L | SC |
| Lim 2011 | H | H | SC | H | L | L | L | SC |
| Kalas 2012 | H | SC | H | H | L | L | L | SC |
| Pasiali 2014 | H | SC | L | H | H | L | L | SC |
| Paul 2015 | H | SC | H | H | L | L | L | SC |
| Ghasemtabar 2015 | SC | L | L | H | H | H | SC | SC |
| Davis 2016 | H | H | SC | H | L | L | L | SC |
| Yoo 2018 | H | SC | L | H | H | H | L | H |
| **ID** | | | | | | | | |
| Aldridge 1995 | SC | H | L | H | H | H | L | H |
| Duffy and Fuller 2000 | SC | H | SC | H | L | H | L | SC |
| Rainey Perry 2003 | H | H | L | H | H | L | L | H |
| Williams 2012 | H | H | SC | H | H | L | L | H |
| Yang 2016 | H | H | H | H | H | L | L | H |
| Zyga 2018 | H | H | SC | H | H | L | L | H |
| Mendelson 2016 | H | H | L | H | H | L | L | H |
| **ADHD** | | | | | | | | |
| Montello 1998 | H | H | H | H | H | L | L | H |
| Jackson 2003 | H | H | H | H | H | L | L | H |
| Rickson 2006 | H | L | H | H | H | L | L | H |
| Gooding 2011 | H | H | SC | H | H | L | L | H |
| Rothmann 2014 | SC | H | L | H | H | L | L | SC |
| **Communication/oral and written language disorders** | | | | | | | | |
| Overy 2003 | H | SC | SC | H | H | L | L | SC |
| Groβ 2010 | H | H | L | H | L | L | L | SC |
| Habib 2016 | H | H | L | H | H | L | L | H |

*Note*: L : Low risk of bias; SC : Some concerns; H : High risk of bias; ROBINS-I: Risk Of Bias In Non-randomized Studies - of Interventions

**Table S2.** Risk of bias in randomized studies using the RoB2 scale

| **Authors** | **Risk of bias arising from the randomization process** | **Risk of bias due to deviations from the intended intervention** | **Risk of bias in measurement of the outcome** | **Missing outcome data** | **Risk of bias in selection of the reported result** | **Overall risk of bias** |
| --- | --- | --- | --- | --- | --- | --- |
| **ASD** | | | | | | |
| Farmer 2003 | SC | H | H | L | L | H |
| Lim 2010 | SC | H | L | L | L | H |
| Lanovaz 2011 | H | H | H | L | L | H |
| Gattino 2011 | L | H | L | L | L | H |
| Sandiford 2013 | SC | H | H | H | L | H |
| LaGasse 2014 | L | H | SC | H | L | SC |
| Thompson 2014 | SC | SC | SC | L | L | SC |
| Porter 2016 | SC | H | SC | L | L | SC |
| Bieleninik 2017 | L | SC | L | L | L | L |
| Sharda 2018 | L | H | L | SC | L | SC |
| Rabeyron 2019 | L | H | L | SC | L | SC |
| Cibrian 2020 | SC | H | SC | L | L | H |
| **Communication/oral and written language disorders** | | | | | | |
| Register 2007 | H | H | H | H | L | H |
| Flaugnacco 2015 | SC | H | H | L | L | H |

*Note*: RoB2: Revised Cochrane risk-of-bias tool for randomized trials
